# Generative adversarial network based on chaotic time series

**DOI:** 10.1038/s41598-019-49397-2

**Published:** 2019-09-10

**Authors:** Makoto Naruse, Takashi Matsubara, Nicolas Chauvet, Kazutaka Kanno, Tianyu Yang, Atsushi Uchida

**Affiliations:** 1 Department of Information Physics and Computing, Graduate School of Information Science and Technology, The University of Tokyo, 7-3-1 Hongo, Bunkyo-ku, Tokyo 113-8656 Japan; 2 Department of Mathematical Engineering and Information Physics, Faculty of Engineering, The University of Tokyo, 7-3-1 Hongo, Bunkyo-ku, Tokyo 113-8656 Japan; 3 Department of Computational Science, Graduate School of System Informatics, Kobe University, 1-1 Rokkodai, Nada, Kobe, Hyogo 657-8501 Japan; 4 Department of Information and Computer Sciences, Saitama University, 255 Shimo-Okubo, Sakura-ku, Saitama City, Saitama 338-8570 Japan

**Keywords:** Computational science, Information technology, Fibre optics and optical communications

## Abstract

Generative adversarial networks (GANs) are becoming increasingly important in the artificial construction of natural images and related functionalities, wherein two types of networks called generators and discriminators evolve through adversarial mechanisms. Using deep convolutional neural networks and related techniques, high-resolution and highly realistic scenes, human faces, etc. have been generated. GANs generally require large amounts of genuine training data sets, as well as vast amounts of pseudorandom numbers. In this study, we utilized chaotic time series generated experimentally by semiconductor lasers for the latent variables of a GAN, whereby the inherent nature of chaos could be reflected or transformed into the generated output data. We show that the similarity in proximity, which describes the robustness of the generated images with respect to minute changes in the input latent variables, is enhanced, while the versatility overall is not severely degraded. Furthermore, we demonstrate that the surrogate chaos time series eliminates the signature of the generated images that is originally observed corresponding to the negative autocorrelation inherent in the chaos sequence. We also address the effects of utilizing chaotic time series to retrieve images from the trained generator.

## Introduction

**Figure 1:**
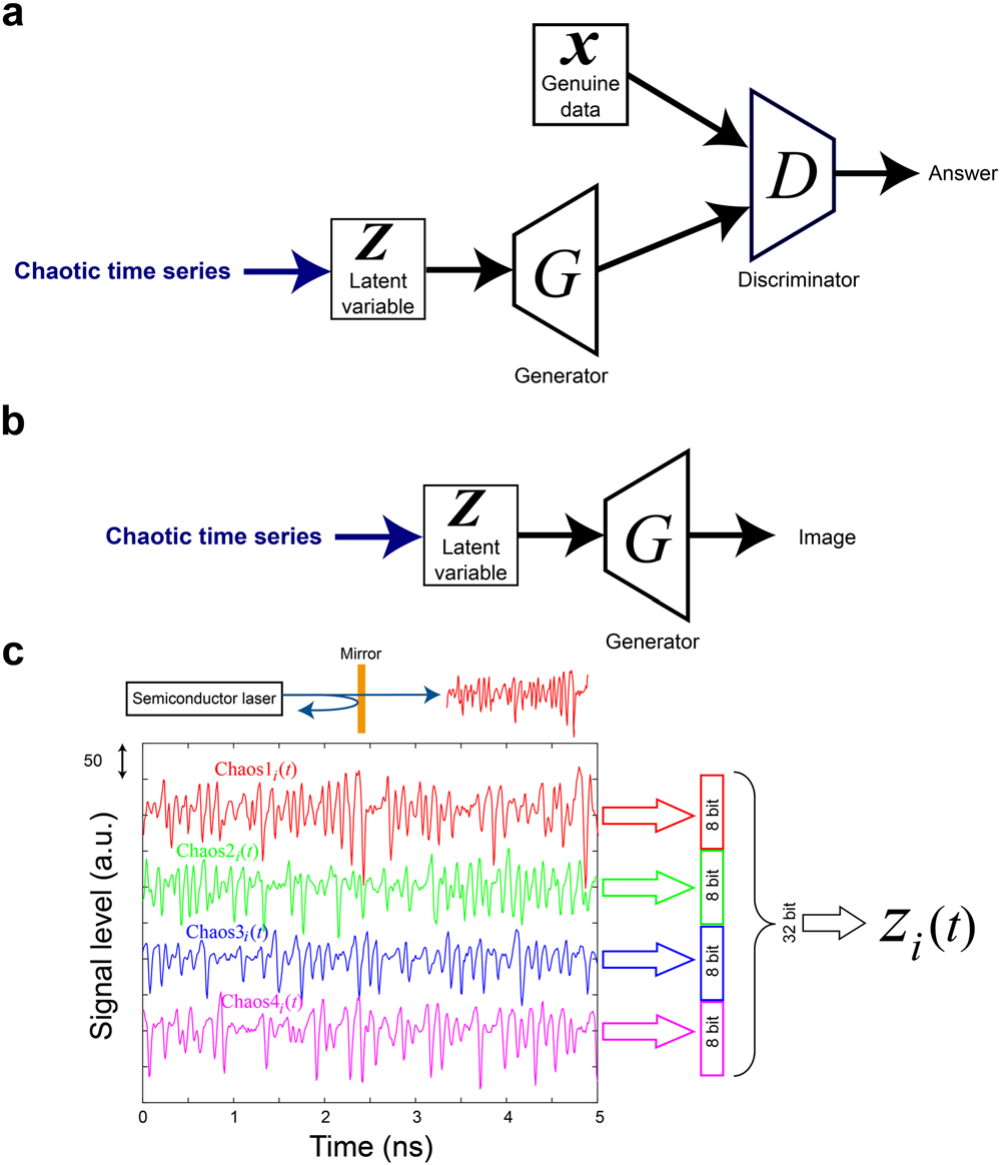
Use of chaotic sequences for GAN. (**a**) Use of chaotic time series in the training phase and (**b**) final data generation in the GAN. (**c**) Construction of latent variables from experimentally generated laser chaos sequences.

Generative adversarial networks (GANs) are becoming increasingly important in the artificial construction of realistic images and related functionalities^1–8^. GANs are based on two types of networks called generators and discriminators, which are denoted by ***G*** and ***D***, respectively, in Fig. 1a and evolve through adversarial learning^1^. Using deep convolutional GANs (DCGANs)^2^ and related types of networks such as progressively growing GANs (PGGANs)^3^, amazingly realistic, high-resolution scenes and human faces have been successfully produced. The generator learns a map from the latent space to the data space over which the given samples are distributed, and the discriminator evaluates the map. Since the objective function is defined as the expected value of the discriminator output over the distribution of latent variables, the training requires vast amounts of pseudorandom numbers for Monte Carlo sampling of the latent variables. In this study, we examined GANs from a physics perspective, considering the effects of utilizing chaotic time series^9,10^ for GANs in the latent space.

Again, while a GAN generally requires large and genuine training datasets, represented by ***x*** in Fig. 1a, vast amounts of pseudorandom numbers called latent variables are also required for the noise source with respect to the generator, as represented by ***Z*** in Fig. 1a. In this study, we utilized chaotic time series that were experimentally generated by semiconductor lasers for the noise source^9–12^ of the GAN. Also, after finishing the learning phase, we examined the effects of utilizing chaotic time series subjected to the trained generator ***G*** as the latent variables to retrieve output images (Fig. 1b). We expect that the inherent nature of chaos, such as its time-domain correlations as well as irregularity, is transformed into the characteristics of the generated images. We show that the similarity in proximity, which describes the robustness with a minute change in the input latent variables, is enhanced, while the overall versatility is not severely degraded. The effects of using chaotic sequences instead of conventional pseudorandom numbers to retrieve output images from the trained generator are also discussed.

It should be noted that ultrafast optical random number generation (RNG) by chaotic lasers^11,12^ was not examined in this study; rather, the primary objective of the present research was to highlight the effects of utilizing experimentally observed chaotic sequences generated by lasers themselves^9,10^ for GAN applications. The utilization of lasers for generating chaos was motivated by their high-speed operations described in the literature^9–12^, which have recently been applied in photonic reservoir computing^13,14^ and decision making^15,16^. Further discussion is provided at the end of the paper.

## Results

The GAN architecture utilized in this study was a DCGAN^2^ with an input image size of 64 × 64. The genuine data sets used for training were 202,559 kinds of human face images available from CelebA^8^. The network structures of the generator ***G*** and discriminator ***D*** as well as the associated parameters are the same as those shown in ref.^2^; for example, in the generator, a series of fractionally strided convolutions is followed by conversion into a 64 × 64 pixel image. A stochastic gradient descent method with a mini-batch size of 128 was used for training. The slope of the LeakyReLU was 0.2. An Adam optimizer^17^ with a learning rate of 0.0002 was used.

### Chaotic sequences

The input vector ***Z*** subjected to generator ***G*** consists of 100 elements of 32 bit floating point numbers between −1 and 1 denoted by ***Z*** = (*Z*_1_, …, *Z*_*i*_, …, *Z*_100_), where *i* = 1, …, 100. Unlike conventional uniformly distributed pseudorandom numbers, ***Z*** is constructed based on experimentally observed chaotic laser time series. A schematic illustration of chaotic time series generation is shown in the upper part of Fig. 1c, where a fraction of the output light from the semiconductor laser is fed back into the cavity of the laser, accompanied by a certain time delay, leading to chaotic oscillation of the laser^9,10^. Indeed, by exploiting the high-bandwidth attributes of light, ultrahigh-speed RNG has been demonstrated in the literature based on the chaotic dynamics of semiconductor lasers^11,12^. However, it should be emphasized that, as mentioned in the *Introduction* section, while the direct physical adaptation of optical ultrafast RNG to deep learning signal processing hardware platforms may be of great interest in the future, the present report does *not* discuss optical RNG. In this study, we adapted the original laser chaos sequences, which were acquired separately prior to the GAN computation process, to examine the qualitative impacts of chaos in the GAN. Related topics are discussed at the end of the paper.

Figure 1c provides examples of chaotic signals, where the four kinds of trains shown, referred to as Chaos 1–4, were acquired experimentally by slightly varying the reflection of the external mirror. The details are provided in the *Methods* section. Chaos 1–4 were sampled by a high-speed digital oscilloscope at a rate of 100 Gsamples/s (10 ps sampling interval (SI)) with 10,000,000 (=10 M) points with an 8-bit resolution. These 10 M data points were stored 100 times for each signal train; hence, there were 100 kinds of 10M-long sequences, denoted as Chaos1_*i*_(*t*), Chaos2_*i*_(*t*), Chaos3_*i*_(*t*), and Chaos4_*i*_(*t*) for the *t*-th sample of all 10 M points during the *i*-th measurement (*i* = 1, …, 100).

We horizontally concatenated Chaos1_*i*_(*t*) to Chaos4_*i*_(*t*) to obtain a 32-bit integer variable by regarding each of the 8-bit values from ChaosX_*i*_(*t*) as an unsigned integer. Each value was then divided by 2^32^, followed by normalization to obtain a range of values from −1 to 1. The resulting values are referred to as *Z*_*i*_(*t*), where *i* ranges from 1 to 100 and *t* spans from 1 to 10^7^.

**Figure 2:**
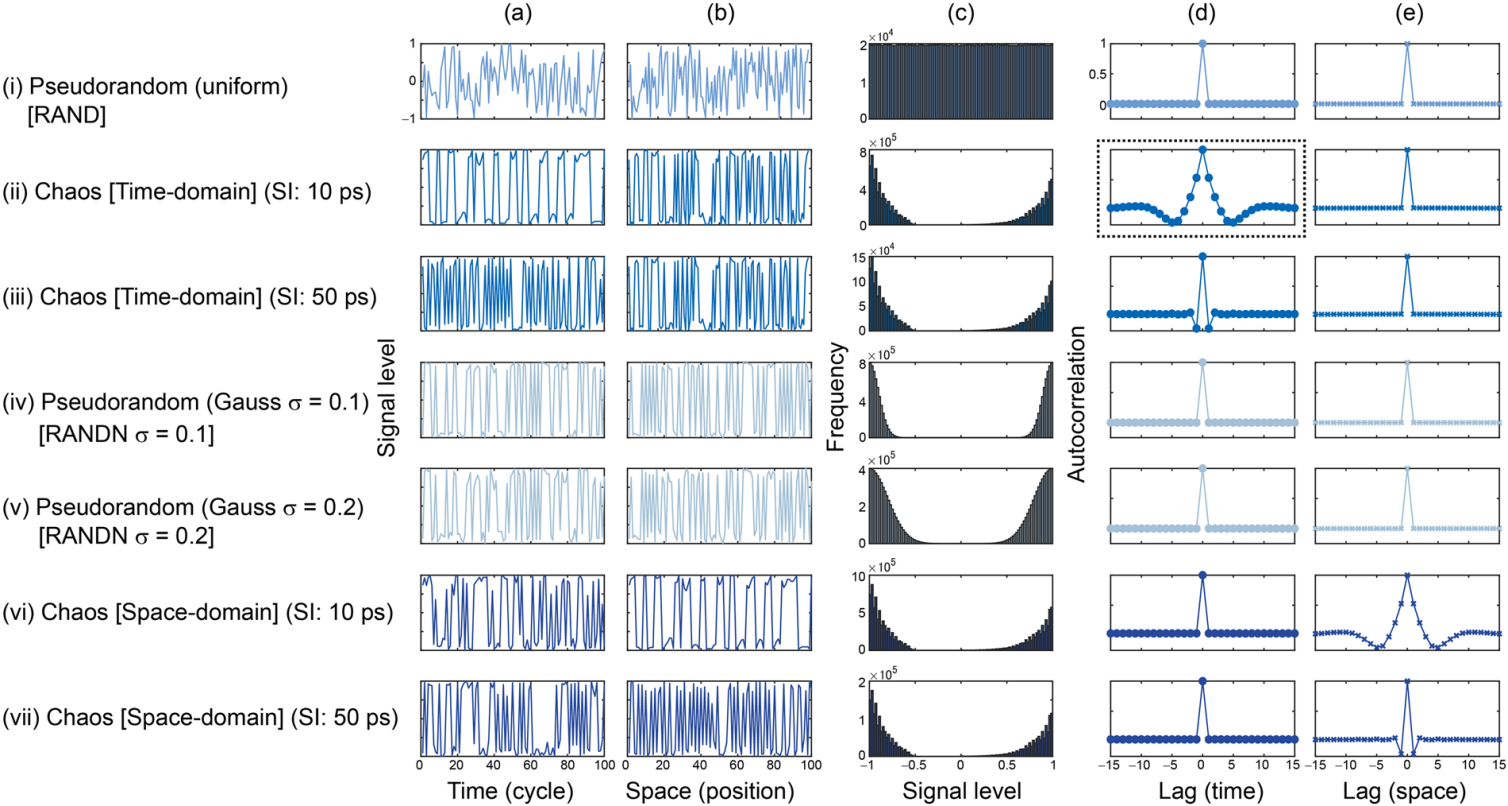
Random sequences for GAN. (i) Pseudorandom numbers. Chaos sequences arranged in the time domain with SI of (ii) 10 ps and (iii) 10 ps. Normally distributed pseudorandom numbers with standard deviations of (iv) 0.1 and (v) 0.2. Chaos sequences arranged in the space domain with SI of (vi) 10 ps and (vii) 50 ps. Signal-level profiles in (**a**) time and (**b**) space domains. (**c**) Signal level histogram. Autocorrelation in (**d**) time and (**e**) space domains.

In the present study, we examined seven kinds of sequences for the latent variable ***Z*** for the GAN. The first consisted of uniformly distributed pseudorandom numbers generated using Mersenne Twister, which are referred to as RAND hereafter. Figure 2(i,a) displays the first 100 points of time sequence *Z*_1_(*i*) (*time*-domain snapshot), while Fig. 2(i,b) represents the array of *Z*_*i*_ as a function of the index *i* and is called a *space*-domain snapshot of ***Z***(*t*). The histogram of the signal level is shown in Fig. 2(i,c), confirming the uniformity of the distribution. Figure 2d,e demonstrate the autocorrelation of ***Z***(*t*) in the temporal and spatial dimensions, respectively. Specifically, Fig. 2d represents the temporal average of the autocorrelation of *Z*_*i*_(*t*) with respect to *t*, whereas Fig. 2e displays the sequence average of the autocorrelation of *Z*_*i*_(*t*) with respect to *i* from 1 to 100. Clearly, since no temporal or spatial correlations are inherent in RAND, the autocorrelation is zero in both Fig. 2(i,d,i,e) except when the lag is zero.

With the chaotic sequence ***Z***(*t*), the temporal and spatial snapshots and the signal level histogram are shown respectively in Fig. 2(ii,a,ii,b,ii,c). The signal incidence exhibits peak levels around −1 and 1 because, as discussed in detail later, the signal level distribution of the original chaotic lasers is similar to a Gaussian distribution with an average of zero. In the above-mentioned construction of ***Z***(*t*), however, we dealt with the 8-bit binary sequences as unsigned integer values; hence, the Gaussian-like distribution was split between positive and negative values with peaks at the edges (−1 and 1). The autocorrelation in the temporal dimension (Fig. 2(ii,d)) does exhibit non-zero values along the time lag; in particular, it shows a negative maximum of around −0.1 when the time lag is −5 or 5. Conversely, the autocorrelation in the spatial dimension (Fig. 2(ii,e)) is zero, demonstrating that there are no correlations among the different elements of ***Z***. Since the sampling rate of the original chaotic lasers is 100 Gsample/s, the signals in row (ii) are referred to as ‘Chaos [Time-domain] (SI: 10 ps)’ hereafter.

As can be seen in the time-domain autocorrelation (Fig. 2(ii,d)), the time difference of 5 cycles, corresponding to the 50 ps SI of the chaotic lasers, is a characteristic variable since it yields the maximum negative autocorrelation. Column (iii) of Fig. 2, entitled ‘Chaos [Time-domain] (SI: 50 ps)’, corresponds to the case in which ***Z***(*t*) was resampled every 50 ps. While the shape of the histogram (Fig. 2(iii,c)) is the same as that in the previous case (Fig. 2(ii,c)), the time-domain autocorrelation shows negative maxima at time lags of −1 and 1.

The data shown in the rows (iv) and (v) are normally distributed random variables with standard deviations of 0.1 and 0.2, referred to as ‘RANDN σ = 0.1’ and ‘RANDN σ = 0.2’, respectively, hereafter. To obtain distributions resembling those of Chaos (ii) and (iii), the positive and negative parts were shifted toward the edges of −1 and 1, which is a transformation equivalent to that conducted to obtain Chaos (ii) and (iii) above. The signal level distribution is similar to the case of chaos when σ = 0.2.

We prepared another arrangement of latent variables from chaos so that the correlations were transformed into the *space* domain of ***Z***(*t*), instead of the *time* domain arrangement represented by the assignment of chaotic sequences *Z*_1_(*t*), *Z*_2_(*t*), …, Z_100_(*t*) to ***Z***(*t*) ($=[{Z}_{1}(t),\cdots ,{Z}_{100}(t)]$). In the case of space domain assignment, the first and second components of ***Z***(*t*), for example, are ${\boldsymbol{Z}}(1)=[{Z}_{1}(1),\cdots ,{Z}_{1}(100)]$ and ${\boldsymbol{Z}}(2)=[{Z}_{1}(101),\cdots ,{Z}_{1}(200)]$, respectively, meaning that ***Z*** consists of consecutive sources of the same sequence (in this case,${Z}_{1}$). Therefore, the correlation among the elements *within* a single ${\boldsymbol{Z}}(t)$ appears. Generally, ***Z***(*t*) is given by

1 $${\boldsymbol{Z}}(t)=[{Z}_{1}(100\times (t-1)+1),\cdots ,{Z}_{1}(100\times (t-1)+100)],$$

when *t* ranges from 1 to 10^5^, recalling that 10^5^ is the number of data points of the original chaotic sequence. Afterwards, for $t={10}^{5}+1$ to $t=2\times {10}^{5}+1$, the second sequence *Z*_2_(*t*) was utilized for ***Z***(*t*):

2 $${\boldsymbol{Z}}(t)=[{Z}_{2}(100\times (t-{10}^{5}-1)+1),\cdots ,{Z}_{2}(100\times (t-{10}^{5}-1)+100)].$$

In this manner, *Z*_3_(*t*), …, *Z*_100_(*t*) were subsequently allocated to ***Z***(*t*). In the histogram equivalent to those of the former time-domain cases, the autocorrelation exhibits contrasting properties; the space-domain autocorrelation (Fig. 2(vi,e)) emerges, while the time-domain one diminishes (Fig. 2(vi,d)). Row (vii) displays another space-domain arrangement obtained by setting the SI to 50 ps, which is referred to as ‘Chaos [Space-domain] (SI: 50 ps)’.

In the training phase, the number of iterations for an epoch was 2000. We examined the output of the resulting model of the generator after 20 epochs. The hardware used for this study was a personal computer environment (HPC Systems, CPU: Intel Xeon 3.0 GHz, RAM: 384 GB, Windows 10) with a single graphical processing unit (ELSA GeForce GTX 1050 Ti 4GB SP).

## Discussion

### Similarity in proximity

**Figure 3:**
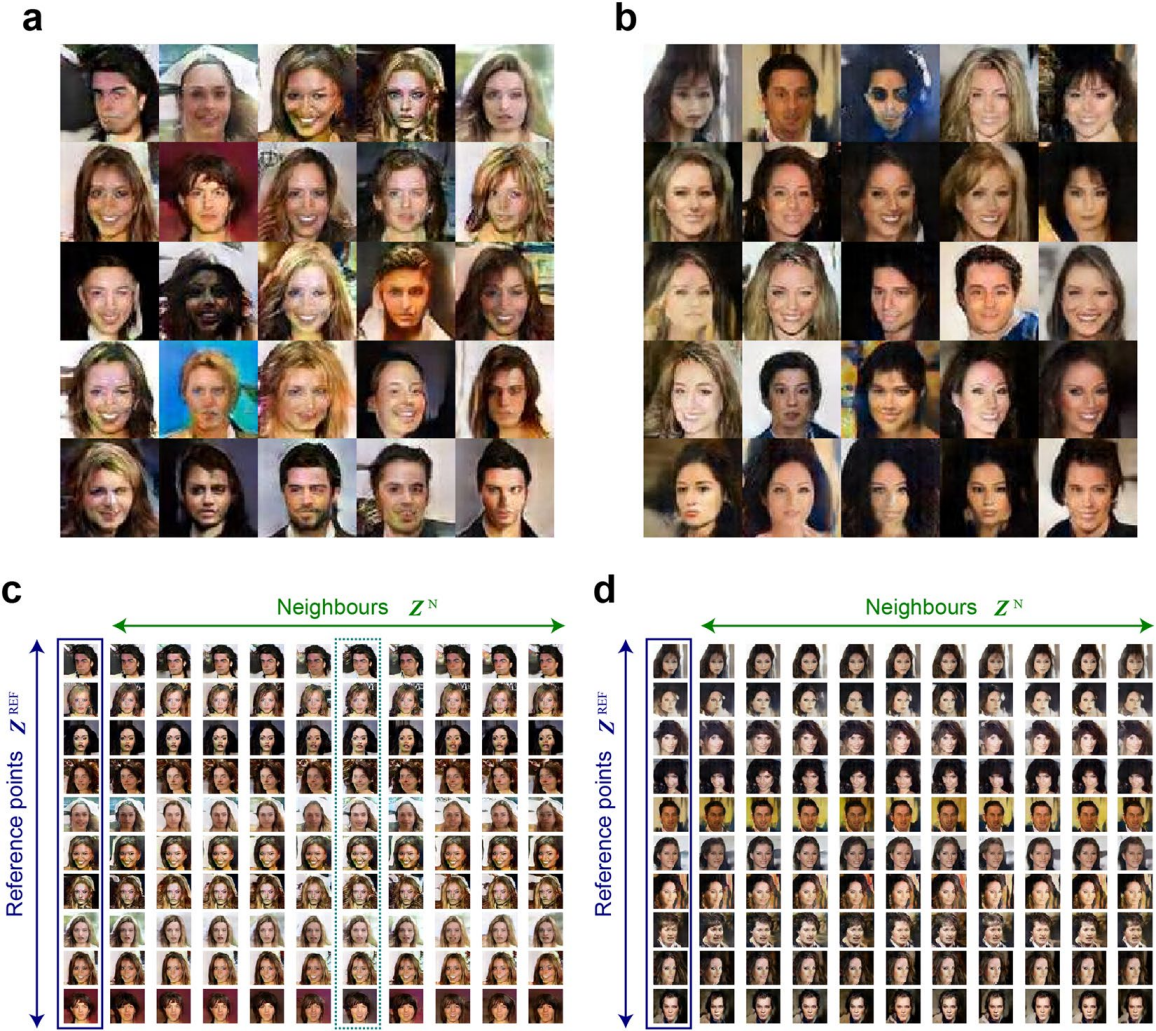
Comparison of images generated using pseudorandom numbers and chaos. Examples of face images generated with training using (**a**) pseudorandom numbers and (**b**) chaotic sequences. Proximity analysis of images generated with training using (**c**) pseudorandom numbers and (**d**) chaos. In generating those images, CelebA datasets^8^ were utilized.

Figure 3a,b show representative examples of the pictures generated using (i) RAND and (iii) Chaos [Time-domain] SI: 50 ps, respectively, where equivalently natural human faces are observable. There are certainly artefacts and unrealistic portions in the images, although the latest sophisticated GAN techniques, such as those described in refs^6,7^, can resolve such problems in the quality of the generated pictures. However, we proceeded with the generation and analysis based on the above-described DCGAN technique and our standard computing environment since the primary objective of the present study was to examine the effects of chaotic sequences.

First, we examined the robustness of the resulting images with respect to slight changes of the input latent variables. With randomly chosen reference points for the latent variables ${{\boldsymbol{Z}}}^{{\rm{REF}}(k)}=({Z}_{1}^{{\rm{REF}}(k)},\ldots ,{Z}_{100}^{{\rm{REF}}(k)})$, where ${Z}_{i}^{{\rm{REF}}(k)}$ ($i=1,\ldots ,100$) was generated using pseudorandom numbers, the reference output picture ${{\boldsymbol{P}}}^{(k)}$ was obtained. In total, *K* = 200 reference points of ${{\boldsymbol{Z}}}^{{\rm{REF}}(k)}$ were chosen, among which 10 cases are displayed in the outlined left-most column (Fig. 3c,d). With respect to a specific ${{\boldsymbol{Z}}}^{{\rm{REF}}(k)}$, its proximity or neighbours are specified by ${{\boldsymbol{Z}}}^{{\rm{N}}(k,l)}=({Z}_{1}^{{\rm{REF}}(k)},\cdots ,{Z}_{90}^{{\rm{REF}}(k)},{Z}_{91}^{{\rm{N}}(l)},\cdots ,{Z}_{100}^{{\rm{N}}(l)})$, where the first 90 elements are the same as those of ${{\boldsymbol{Z}}}^{{\rm{REF}}(k)}$, whereas the last 10 elements were randomly obtained using pseudorandom numbers. Such neighbours are arranged for *L* = 100 points; 10 such neighbouring pictures, ${{\boldsymbol{P}}}^{N(k,l)}$, are shown in the horizontal directions in Fig. 3c,d. The neighbours do not differ much from the reference images, but some of the faces are rather significantly altered in the case of RAND, such as those in the sixth column framed by the dotted green box in Fig. 3c. For quantitative analysis, Pearson’s correlation coefficient between the reference image ${P}^{{\rm{REF}}(k)}$ and ${P}^{{\rm{N}}(k,l)}$ was evaluated using

3 $$R({P}^{{\rm{REF}}(k)},{P}^{{\rm{N}}(k,l)})=\frac{\sum _{x}\sum _{y}({P}^{{\rm{REF}}(k)}-\overline{{P}^{{\rm{REF}}(k)}})({P}^{{\rm{N}}(k,l)}-\overline{{P}^{{\rm{N}}(k,l)}})}{\sqrt{(\sum _{x}\sum _{y}{({P}^{{\rm{REF}}(k)}-\overline{{P}^{{\rm{REF}}(k)}})}^{2})(\sum _{x}\sum _{y}{({P}^{{\rm{N}}(k,l)}-\overline{{P}^{{\rm{N}}(k,l)}})}^{2})}},$$

where *x* and *y* denote the horizontal and vertical axes of the generated images, respectively. The similarity in proximity was characterized using the average over the neighbours and reference points given by

4 $$\frac{1}{KL}\mathop{\sum }\limits_{k=1}^{K}\mathop{\sum }\limits_{l=1}^{L}R({P}^{{\rm{REF}}(k)},{P}^{{\rm{N}}(k,l)}).$$

**Figure 4:**
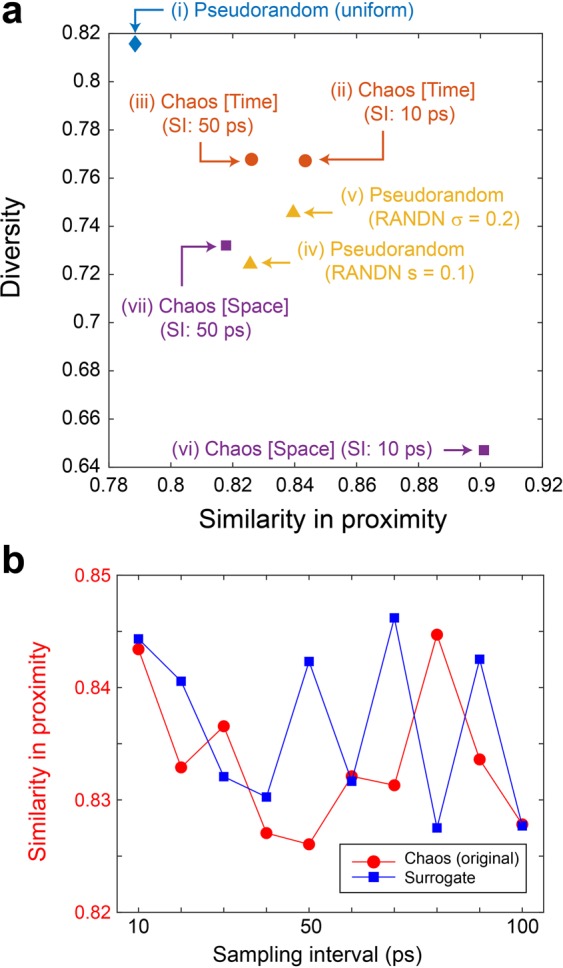
Analysis of the generated images. (**a**) Scatter plot of the similarity of the generated images upon a slight change in the latent variable, called the similarity in proximity, and the diversity of the generated images overall. (**b**) Similarity in proximity as a function of the SI of the laser chaos sequences. The minimum peak value occurs at 50 ps, which corresponds to the maximum negative autocorrelation at 50 ps in the original laser chaos (see Fig. 2). With the surrogate chaos sequences, obtained by eliminating the temporal structure from the original chaos sequence, no such tendency is observable.

Figure 4a summarizes the values of the similarity in proximity of the images generated via the seven kinds of random sequences [(i)–(vii) in Fig. 2]. With the use of chaos, the similarity in proximity is enhanced, particularly for the space-domain chaotic sequences (vi), compared with the conventional uniformly distributed random numbers (i). The enhanced similarity in proximity is, however, also observable when RANDN with σ = 0.2 was used (v), indicating that the *distribution* of the input ***Z*** affects the resulting properties, rather than the chaotic nature of the random sequences. This point will be discussed further in the following sections.

### Diversity

We quantified the diversity of the generated images by employing the multi-scale structural similarity (MS-SSIM)^18^, which has been applied in the analysis of images generated by GANs in the literature^19,20^. The MS-SSIM is a multi-scale variant of perceptual similarity that has been shown to correlate well with human judgement^18^. The value of the MS-SSIM ranges from 0 (low similarity) to 1 (high similarity). After training the generator for the seven different random sources, 10,000 images were produced using pseudorandom numbers. We calculated the MS-SSIM scores between 1,000 randomly selected pairs of images. Here we define *diversity* as [1 − *S*], where *S* is the average of all of the MS-SSIM values. The figure-of-merits were examined by taking the averages of 10 different trials (see the *Methods* section for details).

Intuitively, high similarity in proximity corresponds to decreased global diversity, while low similarity in proximity indicates higher global diversity. Indeed, as summarized in Fig. 4, the maximum and minimum diversity are given by (i) RAND and (vi) Chaos [Space-domain] (SI: 10 ps), which coincide with the minimum and maximum similarity in proximity, respectively. However, such correspondence does *not* necessarily hold with the time-domain chaos; the similarity in proximity of time-domain chaos (ii) is the second largest, but the diversity of (ii) is also large (the third largest, almost the second largest). That is, the global diversity is not severely damaged in the case of chaos. These observations suggest that the properties inherent in the original random sequences affect the resulting generated images.

To examine such properties further, the SIs of the original laser chaos sequences were configured from 10 ps to 100 ps in 10 ps increments, and each sequence was then configured as the latent variable ***Z***(*t*) via the time-domain arrangement introduced above. The generated images after training were subjected to similarity in proximity analysis. Again, 10 trials were conducted for each data set, and their average was calculated. Recall that, as shown in the dotted box in Fig. 2(ii,d), the time-domain correlation of ***Z***(*t*) has its maximum negative peak value when the time lag is 50 ps. As demonstrated by the square marks in Fig. 4b, the similarity in proximity also shows a peak value at an SI of 50 ps. Meanwhile, the circular marks in Fig. 4b represent the similarity in proximity upon randomly shuffling the input chaotic sequences to eliminate the temporal structure contained in the original chaos (see the *Methods* section for details regarding the surrogate time series). Consequently, the similarity in proximity obtained using the surrogate chaos series exhibits an uncertain profile since the surrogation removes the original temporal structure; this observation supports the transformation of the time-domain property of the input random sequences into the generated images. These results indicate that certain characteristics of random sequences used in the data generation phase, e.g., nonlinearity in chaos, can potentially be inferred from the generated images as a kind of signature, which implies some relation to the security aspects of GANs^21,22^. Such points will be addressed in future studies.

### Retrieval by chaos

We also generated output images using the trained generator ***G*** with chaotic sequences (time-domain arrangement, 50 ps interval) as well as normally distributed pseudorandom numbers with a standard deviation of 0.2 (RANDN), unlike the conventional uniformly distributed pseudorandom numbers (RAND).

**Figure 5:**
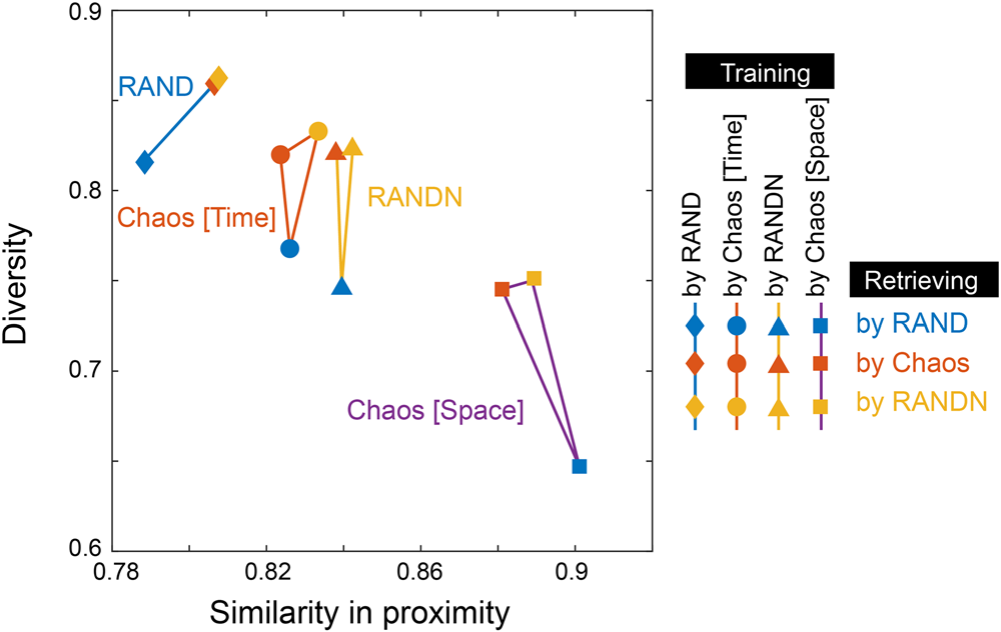
Analysis of the generator trained using chaos. Similarity in proximity and diversity when the output images were obtained from the generators trained using pseudorandom numbers and chaos.

In Fig. 5, the random sequences used for training are represented by different *shapes* of marks: diamonds for uniformly distributed pseudorandom numbers (RAND), circles for time-domain chaos (SI: 50 ps), triangles for nonuniformly distributed pseudorandom numbers (RANDN σ = 0.2), and squares for space-domain chaos (SI: 10 ps). From these generated models, we retrieved images by using different types of random sequences, which are indicated by different *colours*: blue for RAND, orange for chaos, and yellow for RANDN. When the generator was trained using RAND, both the proximity similarity and diversity were enhanced when the generator retrieval was performed using chaos or RANDN rather than RAND. When the generator was trained using chaos or RANDN, the diversity increased when chaos or RANDN was employed instead of RAND. At the same time, no difference between chaos- and RANDN-based retrieval is evident, indicating that the distribution of the random numbers is the primary factor producing such an effect. Indeed, the average of the minimum Hamming distances between the latent variables is about 3 in the case of RAND, while those in the cases of chaos and RANDN are about 10, which would lead to superior diversity of the images generated by chaos and RANDN. Further study is necessary to improve understanding of the underlying mechanisms that distinguish chaos and RANDN in the retrieval phase.

### Platform of chaos

Before concluding, we briefly review the context of the present study and related topics for future research regarding chaotic GANs. In this paper, we highlighted the utilization of experimentally observed, laser-generated chaotic time series in GANs, whereby the inherent time-domain correlations and ultrahigh-speed operation abilities were exploited. Recent advances in photonic device technologies may allow systems to be implemented compactly using integrated^23^ and silicon photonics^24^.

Other physical processes could also be considered for generating chaotic time series, especially in cases in which ultrahigh operating speed is not required, such as in simple electrical circuits^25,26^, microelectro-mechanical systems, etc.^27^. In optical domains, various methods of generating chaotic dynamics other than the delayed optical feedback employed in the present study are known^28^, such as spontaneous mode switching in ring lasers^29^, mode competition in vertical-cavity surface-emitting lasers^30^, and light–atom coupling^31,32^. Examining the compositions of these physical platforms and GANs is a topic for future study. Furthermore, there are various theories of chaotic time series ranging from discrete maps (e.g., Lorentz maps) to nonlinear ordinary differential equations (e.g., the Rössler equation)^33^. Chaotic lasers with delayed feedback are known to be well characterized by the Lang-Kobayashi equation^9,10^. Furthermore, a thorough analysis between chaos and other irregular time series such as coloured noise and higher-order correlation noise^34^ in GANs will be of interests. In the literature of other computing applications, comparable performances have been reported in photonic reservoir computing between chaotic lasers and coloured noise^35^ while chaotic sequences outperform coloured noise in decision making^16^. Theoretical insight into how GAN systems work with respect to these different types of chaotic dynamics could be the focus of additional future studies.

## Conclusion

We examined the effects of chaotic sequences for artificial data generation by developing a DCGAN with CelebA datasets in which experimentally observed laser chaos signals were utilized for the latent variables. The inherent properties of the chaotic signals, such as temporal correlations, were transformed into the generated data observed by performing similarity and diversity analysis of the generated data, which are also supported by the comparisons to the cases with surrogate chaotic sequences. This study is a first step toward gaining insight into the intersection between chaos and GANs, or in a wider context, an initial exploration of novel composite systems including artificial intelligence, nonlinear dynamics, and photonics technologies.

## Methods

### Laser chaos

A semiconductor laser (NTT Electronics, KELD1C5GAAA) operated at a centre wavelength of 1547.785 nm was coupled with a polarization-maintaining (PM) coupler. The light was connected to a variable fibre reflector, which provided delayed optical feedback to the laser, generating laser chaos. (i) Chaos 1, (ii) Chaos 2, (iii) Chaos 3, and (iv) Chaos 4 were obtained using the variable reflector by letting 210, 120, 80, and 45 μW of optical power be fed back to the laser, respectively. The length of the fibre between the laser and reflector was 4.55 m, corresponding to a feedback delay time of 43.8 ns. The output light at the other end of the PM coupler was detected by a high-speed, AC-coupled photodetector (New Focus, 1474-A) through an optical isolator and optical attenuator, which was sampled using a digital oscilloscope (Tektronics, DPO73304D).

### Signal processing

The GAN model and parameters are those shown in ref.^2^. The code of the GAN was built based on Chainer^36^. The generated images were analysed using MATLAB with the Signal Processing Toolbox. We utilized the code of MS-SSIM available from ref.^37^. Mersenne Twister was used as the pseudorandom number generator (to obtain RAND and RANDN). In the training phase (or model generation phase), 10 different random sequences were generated using different seeds, which were subjected to independent learning processes. We examined the average of the resulting figures. Regarding the cases with chaotic sequences, ***Z*** was defined as a matrix subjected to the generator, with the rows sequentially used as the latent variables. Here, the *n*-th trial of learning began with row number 128 × (*n* − 1) + 1 of ***Z***. The random shuffling of the chaotic sequences was implemented using the randperm function in MATLAB. The average of 10 different random shufflings was examined for the surrogate data.

## Data Availability

The datasets generated during the current study are available from the corresponding author on reasonable request.
